# Blast Resistance of Confined Multilayer Graded Corrugated-Core Sandwich Cylindrical Shells

**DOI:** 10.3390/ma19010101

**Published:** 2025-12-27

**Authors:** Pengbo Su, Bin Han, Yiyang Zhong, Zeliang Yu, Yonggang Xue, Haiming Liu, Tian Jian Lu

**Affiliations:** 1 Xi’an Institute of Space Radio Technology, Xi’an 710100, China; su_pengbo@126.com (P.S.); jivqa19@126.com (Y.Z.); yuzeliang715@hotmail.com (Z.Y.); 18092166275@163.com (Y.X.); liuhaiming1979@126.com (H.L.); 2 School of Mechanical Engineering, Xi’an Jiaotong University, Xi’an 710049, China; 3 State Key Laboratory of Mechanics and Control of Mechanical Structures, Nanjing University of Aeronautics and Astronautics, Nanjing 210016, China; tjlu@nuaa.edu.cn; 4 MIIT Key Laboratory of Multifunctional Lightweight Materials and Structures, Nanjing University of Aeronautics and Astronautics, Nanjing 210016, China

**Keywords:** blast-resistant container, gradient multilayer corrugated sandwich shell, surrogate-based optimization

## Abstract

**Highlights:**

**What are the main findings?**

- Graded multilayer corrugated-core shells enhance resistance to internal blasts..
- An inner-thick/outer-thin wall gradient reduces outer-facesheet deformation by up to 75%.
- Thickness grading performs better than height grading under the studied blast loads.
- Surrogate modeling with ASA optimization identifies optimal thickness distributions.

**What are the implications of the main findings?**

- Aligning core strength with blast attenuation promotes more uniform and complete layer compaction.
- Optimized designs can reduce mass by ~18% at a prescribed deformation limit.
- Alternatively, they can reduce deformation by ~20% at a prescribed mass limit.

**Abstract:**

A graded multilayer corrugated-core sandwich cylindrical shell is proposed as an innovative blast-resistant container to resist internal blast loading. The blast resistance performance of both uniform and graded multilayer corrugated shells was systematically investigated through finite element analysis. Results revealed that sandwich shells featuring an internally thick and externally thin core wall arrangement exhibited superior blast resistance. This configuration optimally aligns with the natural attenuation behavior of blast pressure, which gradually decreases from inner to outer layers during multilayer core collapse. Structures with core layer height gradients, characterized by internally high and externally low layers, also demonstrated enhanced performance under blast loading. While increasing the gradient magnitude generally improves blast resistance, this benefit diminishes with escalating blast intensity. Notably, wall-thickness-graded structures consistently outperformed height-graded configurations. Finally, a radial basis function surrogate model combined with adaptive simulated annealing optimization was employed to identify optimal thickness-graded cylindrical shell configurations tailored for either maximum blast resistance or minimum structural mass.

## 1. Introduction

Protective structures under explosion loads are generally known as blast-resistant containers. These containers are designed to mitigate the impact of explosive shock waves and explosion products. They are widely used in military applications, such as explosion testing, weapon storage, and the disposal of expired weapons and ammunition [1]. Depending on their function and design principle, blast-resistant containers are classified into single-use and reusable types. The single-use type allows plastic deformation, whereas the reusable type must remain elastic throughout its service life [2]. The blast-resistant vessel investigated in this study belongs to the single-use category. As a critical emergency device for isolating, transporting, or detonating explosives, it must be lightweight yet highly blast-resistant.

Research on blast-resistant containers can be categorized into three main areas: (i) the characteristics of explosive loads under confined conditions; (ii) the dynamic response and stress state of the container shells; and (iii) the design, fabrication, and lifetime assessment of blast-resistant containers. Studies related to explosive loads and shell stress states can be found in previous works [3,4]. In the present study, particular emphasis is placed on the structural design and large-deformation response of the shell under internal blast conditions.

Extensive research has been conducted on the implosion behavior of single-walled metal shells. Duffey et al. [5] experimentally investigated the radial deformation of mild steel tubes under implosion and developed a theoretical model to predict the deformation. Based on experimental results, Benham et al. [6] reported that, in addition to the initial explosive pulse, the accumulation of gas pressure within a confined container also contributes to the radial deformation of metal cylindrical shells, and they established a corresponding theoretical model. Rushton et al. [7] studied the failure modes of seamless steel tubes under implosive loading through experiments, simulations, and theoretical analysis, finding that cylindrical charges induce greater wall deformation than spherical charges. Langdon et al. [8] and Ozinsky [9] employed experiments and finite element analysis to examine the final radial deformation of 304 stainless steel tubes subjected to centrally and end-detonated PE4 charges of different equivalent masses. They found good agreement between finite element results and experimental data when the charge was placed at the tube’s mid-length, and the maximum radial deformation increased linearly with charge mass. In summary, single-walled metal shell structures exhibit stable performance under implosion, and the related research is relatively mature. However, achieving adequate blast resistance typically requires relatively thick shell walls, leading to high structural mass. This limits portability and applicability in confined public spaces such as subways and airports.

To overcome these issues, researchers have proposed sandwich-type shell structures. Shen et al. [10] designed and fabricated an aluminum foam sandwich cylindrical shell and analyzed its dynamic response through experiments and simulations, demonstrating superior performance over a solid shell of equal mass. Karagiozova et al. [11] studied the response of polystyrene foam sandwich cylindrical shells under implosive loading by experiments, simulations, and theoretical analysis, reaching similar conclusions. Liang et al. [12] generated a microscopic model of foam materials based on Voronoi diagrams and performed finite element analyses on double-layer gradient foam sandwich shells to examine the effects of foam gradients and facesheet thickness on blast resistance. They further conducted implosion experiments [13] to analyze the deformation process and protective mechanisms of the sandwich structure. Song et al. [14] proposed a sandwich cylindrical shell with a polyurea core and confirmed its superior blast resistance through implosion experiments. Liu et al. [15] investigated homogeneous and gradient foam sandwich cylindrical shells via finite element analysis, demonstrating that both configurations provided improved blast resistance over equivalent solid-walled shells. In summary, existing studies consistently show that sandwich shells offer significant advantages in blast resistance compared to solid-walled shells. Although most of these works focused on foam cores, a considerable body of literature has also addressed the mechanical performance of foam-core sandwich shells under various quasi-static or dynamic loadings (see Refs. [16,17,18,19] for details), whereas research on periodic lattice-core structures remains relatively limited.

In addition to homogeneous cores, gradient design offers an effective means of enhancing the mechanical performance of sandwich structures. Early studies on gradient configurations mainly focused on foam cores and demonstrated that graded foams undergo greater compression under external loads, thereby achieving superior energy absorption [20,21,22,23,24,25,26,27]. Cui et al. [28] investigated the compressive behavior of foam structures with different gradient configurations under impact loading through finite element analysis. They found that a gradient configuration with foam density decreasing from the impact side to the opposite side provided the best impact resistance, outperforming homogeneous foams. Moreover, increasing the gradient difference further enhanced this advantage. Liu et al. [15] proposed cylindrical sandwich shells with homogeneous and graded foam cores and analyzed their response under internal blast loading using finite element simulations. The results showed that an inner-stiff–outer-soft gradient configuration achieved the best blast resistance, superior to that of homogeneous cores. Beyond foam materials, Li et al. [29] designed a cylindrical shell with graded circular tube cores and numerically examined its blast resistance. They concluded that a gradient structure with tube wall thickness gradually decreasing from the inner to the outer layer exhibited the most favorable blast-resistant performance.

Corrugated structures represent a typical two-dimensional periodic lattice configuration and are widely used as core materials in sandwich panels. They have attracted great attention in recent years because of their high stiffness-to-weight ratio, excellent energy-absorption capability, and ease of fabrication. A number of investigations have clarified their mechanical behavior under static and dynamic loadings. Valdevit et al. [30] and Wadley [31] studied the structural efficiency and multifunctional potential of metallic corrugated cores. Wadley et al. [32] and Xue and Hutchinson [33] examined their impact resistance and load-carrying efficiency, showing that both core geometry and wall thickness strongly affect deformation patterns and energy absorption. Other studies have explored their performance under high-rate or blast loading, and efforts have been made to improve structural crashworthiness by introducing hierarchical or multilayer corrugated configurations [34,35,36]. Researchers have also investigated the use of corrugated cores in sandwich and cylindrical shells. Su et al. [37] experimentally and numerically studied the energy-absorption characteristics of all-metallic corrugated sandwich cylindrical shells under axial compression, revealing superior load-carrying capacity compared with traditional designs. Origami-inspired corrugated metamaterials have also been developed to achieve tunable stiffness or multifunctional performance [38,39,40]. Although these studies have demonstrated the advantages of corrugated cores in improving stiffness, strength, and impact resistance, most prior work has focused on planar panels or axially loaded structures. The implosion behavior of corrugated-core sandwich cylindrical shells under internal blast loading has not been thoroughly investigated.

Building on this concept, the present work aims to investigate the deformation and failure mechanisms of graded multilayer metallic sandwich shells with corrugated cores under internal blast loading. To achieve this goal, a validated finite element model is established based on available experimental data, ensuring the accuracy of the numerical approach. Using this model, a series of parametric studies are performed to investigate the effects of core configuration, gradient design, and material arrangement on the structural response. Furthermore, a surrogate model-based optimization method is employed to determine the optimal gradient configuration that maximizes blast resistance and structural efficiency. Accordingly, this study includes model development and validation, parametric investigations of gradient effects, and surrogate-based optimization for thickness-graded designs.

## 2. Finite Element Model

### 2.1. Modeling Geometry

**Figure 1:**
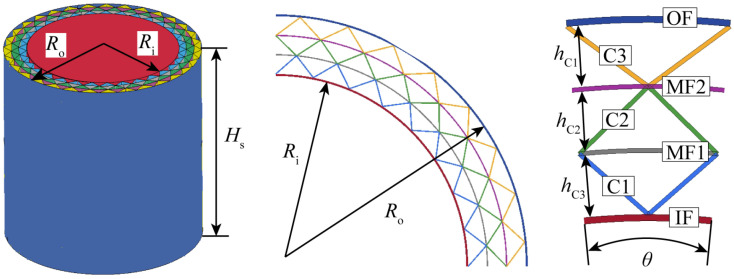
Geometric model of the multilayer corrugated sandwich cylindrical shell.

Figure 1 shows the geometric model of the multilayer corrugated-core sandwich cylindrical shell investigated in this study. The structure consists of an inner and outer facesheet, three layers of corrugated core, and two intermediate facesheets separating them. For clarity, the components are labeled as follows: inner facesheet (IF), the first corrugated core (C1), the first intermediate facesheet (MF1), the second corrugated core (C2), the second intermediate facesheet (MF2), the third corrugated core (C3), and outer facesheet (OF). In Figure 1, the inner radius, outer radius, and total height of the cylindrical shell are defined as $R_{i}$, $R_{o}$, and $H_{s}$, respectively. The heights of the three corrugated core layers are denoted as $h_{c1}$, $h_{c2}$, and ${\;h}_{c3}$, while the central angle corresponding to a single corrugation unit cell is θ. The wall thicknesses of the components are defined as follows: IF thickness $t_{if}$, C1 thickness $t_{c1}$, MF1 thickness $t_{mf1}$, C2 thickness $t_{c2}$, MF2 thickness $t_{mf2}$, and OF thickness $t_{of}$. The thicknesses of MF1 and MF2 are set to the average of their adjacent corrugated core thicknesses, i.e.,$\;t_{mf1}=(t_{c1}+t_{c2})/2$ and $t_{mf2}=(t_{c2}+t_{c3})/2$. For this study, the following parameters are fixed: $R_{i}=300\;\mathrm{mm}$, $R_{o}=390\;\mathrm{mm}$, $H_{s}=800\;\mathrm{mm}$, $t_{if}=3\;\mathrm{mm}$, and $t_{of}=3\;\mathrm{mm}$.

In the Type I (core thickness gradient) design, the wall thicknesses of the three corrugated cores *t*_c1_, *t*_c2_, and *t*_c3_ were varied while keeping the layer heights identical to those of the uniform configuration. The wall thicknesses were set to 3 mm, 2 mm, and 1 mm, denoted as L, M, and S, respectively. This yielded six possible combinations of the three core layers, summarized in Table 1. The prefix “I-” indicates the gradient Type (core thickness gradient), and the subsequent three letters represent the wall thicknesses of the inner, middle, and outer cores, respectively. For example, configuration I-SML corresponds to a structure with inner, middle, and outer core thicknesses of 1 mm (S), 2 mm (M), and 3 mm (L), respectively. Table 1 also lists the masses of the uniform and gradient configurations. As their masses differ only slightly, all structures are considered to have approximately equal mass in subsequent analyses.

The Type II gradient design, referred to as the core height gradient, was achieved by varying the core layer heights ($h_{c1}$–$h_{c3}$) to 40 mm, 30 mm, and 20 mm, denoted T, M, and S, respectively, while maintaining uniform core wall thicknesses. Similar to Type I, this approach produced six possible configurations (e.g., II-SMT), with their nomenclature summarized in Table 2. The masses of these configurations, also presented in Table 2, were found to be nearly identical. Therefore, all structures are considered to have approximately equal mass in subsequent performance comparisons.

### 2.2. Boundary Conditions and Contact Properties

**Figure 2:**
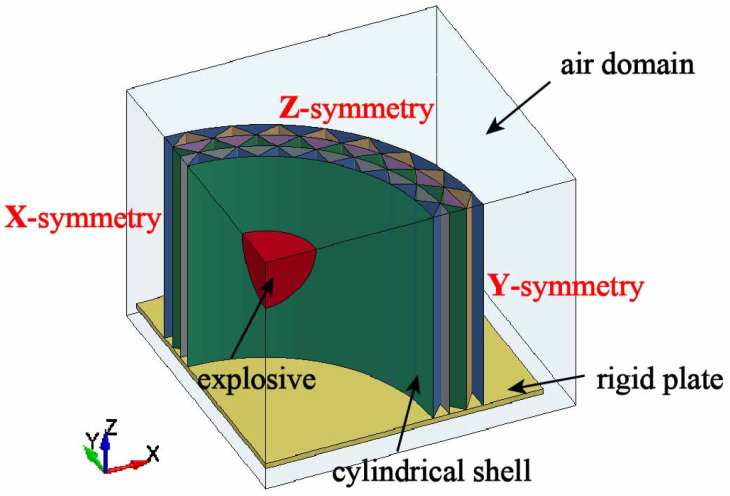
Finite element model for the multilayer corrugated sandwich cylindrical shell subjected to internal blast loading.

This study investigated a sealed multilayer corrugated sandwich cylindrical shell subjected to internal blast loading. The finite element analysis was performed using the explicit dynamics solver LS-DYNA 971 (Livermore, CA, USA). The finite element model, illustrated in Figure 2, consisted of the explosive, the multilayer corrugated sandwich cylindrical shell, a rigid plate, and an air domain. Considering the structural symmetry, boundary conditions, and loading characteristics, a 1/8 segment of the full model was established as the computational domain.

The base of the cylindrical shell was rigidly connected to a stationary end plate, replicating the clamped boundary condition. The detonation and the resulting blast wave were modeled within an enclosed air domain, which completely surrounded the shell and the rigid plate. The expansion of detonation gases, their interaction with surrounding air, and subsequent reflection from the shell wall were simulated through a fluid–structure interaction (FSI) approach. Non-reflecting boundary conditions were prescribed on the outer surfaces of the air domain to prevent artificial wave reflections and ensure physically realistic pressure decay. A spherical PE4 charge was located at the geometric center of the air cavity, representing the explosive. Detonation initiation occurred at its center. The corresponding FSI coupling between the fluid region (air and detonation products) and the solid region (metal shell) ensures momentum and pressure continuity during shock transmission.

### 2.3. Element and Material

The finite element model consisted of the shell structure, rigid plates, explosive charge, and the surrounding air domain. The metallic components (shell and end plate) were modeled using a Lagrangian description, whereas the explosive and air regions adopted an Eulerian description to capture the expansion and flow of detonation products. Together, they formed a three-dimensional Arbitrary Lagrangian–Eulerian (ALE) framework capable of modeling internal blast-induced fluid–structure interaction.

The shell part was meshed with four-node quadrilateral shell elements based on the Belytschko–Tsay formulation (corresponding to SHELL163, ELFORM = 2 in LS-DYNA). An element size of 0.25 mm × 0.25 mm was adopted to provide a good balance between computational accuracy and efficiency. The rigid end plate was discretized with eight-node hexahedral solid elements (equivalent to SOLID164, ELFORM = 1) using a 0.5 mm cubic mesh.

For the fluid domain, the explosive and air regions were both modeled with multi-material ALE solid elements (corresponding to SOLID164, ELFORM = 11 in LS-DYNA). The total number of elements depended on the explosive mass; for example, in the 5 kg charge case, the explosive and air domains contained approximately 6800 and 141,000 elements, respectively. The outer boundaries of the air domain were defined as non-reflecting surfaces to prevent spurious wave reflections and ensure realistic propagation of the blast wave.

**Figure 3:**
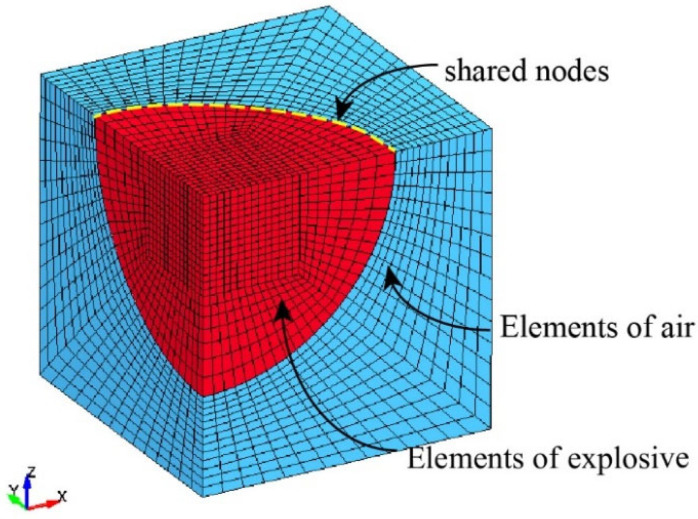
Finite element model of the air and explosive domains.

Figure 3 illustrates the finite element mesh of the air and explosive domains and their spatial relationship with the structural components. The spherical explosive was positioned at the geometric center of the air cavity, and the computational grids were refined radially near the charge to capture steep pressure gradients. The uniform transition of mesh density in the outer air region ensured smooth wave transmission and stable fluid–structure coupling.

Coupling between the explosive and air was achieved through multi-material ALE advection, ensuring pressure and material continuity at their shared interface. The interaction between the Lagrangian solid domains (the metallic shell and end plate) and the Eulerian fluid domains (air and explosive) was handled by a constrained Lagrangian–Eulerian interface algorithm, which accurately transfers transient pressure and momentum loads to the structure. This method is physically equivalent to the *CONSTRAINED_LAGRANGE_IN_SOLID formulation in LS-DYNA.

The metallic materials (corrugated cores and facesheets) were made of 304 stainless steel, whose mechanical behavior under high strain-rate loading was described by the Johnson–Cook constitutive model, which accounts for strain hardening, strain-rate sensitivity, and thermal softening. The corresponding pressure–volume relationship was governed by the Grüneisen equation of state (EOS). In LS-DYNA, these are implemented through the *MAT_JOHNSON_COOK and *EOS_GRUNEISEN definitions. The associated parameters used in this study are provided in Table 3 and Table 4, consistent with the previous literature [8].

The PE4 explosive was modeled as a reacting high explosive with a Jones–Wilkins–Lee (JWL) equation of state, describing the expansion of detonation products and their pressure evolution during the explosion process. This behavior was implemented in LS-DYNA using *MAT_HIGH_EXPLOSIVE_BURN together with EOS_JWL. The adopted density, detonation velocity, Chapman–Jouguet pressure, and empirical JWL constants are listed in Table 5, following Ref. [8].

The surrounding air was represented as a compressible gas described by a linear-polynomial equation of state, characterized by its reference density and polynomial coefficients (*C*_1_–*C*_6_), as shown in Table 6. In LS-DYNA, this corresponds to a *MAT_NULL material with *EOS_LINEAR_POLYNOMIAL, a widely used setup for air blast simulations.

The overall modeling framework described above enables the coupled simulation of detonation initiation, blast wave propagation, and dynamic deformation of the multilayer corrugated-core shell, achieving both numerical stability and physical accuracy.

### 2.4. Validation of the Finite Element Model

To validate the reliability of the numerical model, the present three-dimensional finite element (FE) simulation results were compared both with the experimental measurements and the two-dimensional FE calculations reported by Langdon et al. [8].

**Figure 4:**
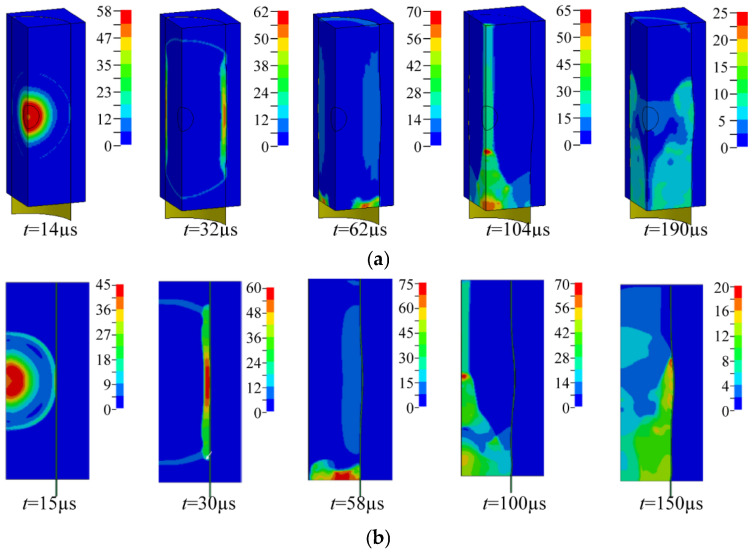
Simulated pressure contour (**a**) 3D FE model in this study; (**b**) 2D FE model from Ref. [8].

Figure 4a shows the pressure contours from the present 3D FE simulation of a 50 g PE4 charge, while Figure 4b presents the 2D FE contours from Ref. [8]. The results are in good agreement. The explosion process is described as follows: After detonation, the pressure wave expanded spherically into the air domain (*t* = 14 μs), reached the cylinder wall and reflected (*t* = 32 μs), then reached the closed end and reflected again (*t* = 62 μs–104 μs). The interaction of these reflected waves produced repeated pressure loading on the cylinder wall (*t* = 190 μs).

**Figure 5:**
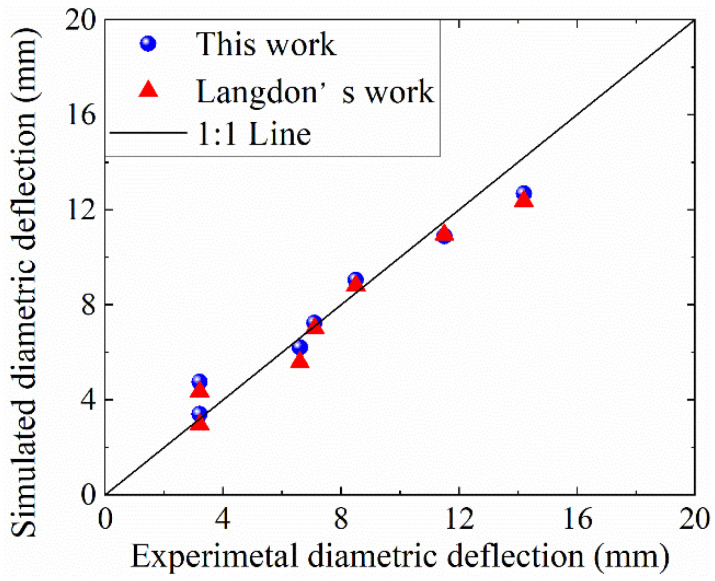
Comparison of simulated and experimental results [8] for the single-walled cylindrical shell internal air-blast loading.

Figure 5 and Table 7 summarize a quantitative comparison of the maximum radial deformations obtained experimentally [8] and those predicted by the present 3D FE model and the previous 2D model for 25–55 g PE4 charges. Overall, the 3D simulation reproduces the experimental deformation magnitudes and trends accurately. For most charge masses, the relative difference is within ±11%. A larger deviation is observed for the 30 g case (≈49%), which is attributed primarily to experimental scatter typically associated with blast testing, together with experimental uncertainty. Because the absolute deformation in this case is very small (3.2 mm), even a minor absolute fluctuation can lead to a disproportionately large percentage difference. Nevertheless, the overall trend and the level of agreement remain satisfactory, indicating that the proposed 3D FE model is suitable for subsequent analyses.

In summary, comparisons with both the experimental data and the 2D FE results [8] verify that the developed three-dimensional FE model accurately captures the essential physical processes: (i) the multi-material flow of the explosive and air, (ii) fluid–structure interaction between the air domain and the cylinder wall, and (iii) dynamic response of the cylinder structure under internal blast loading. Therefore, the model meets the accuracy requirements for evaluating the blast resistance of uniform and graded multilayer corrugated sandwich cylindrical shells.

## 3. Blast Resistance Performance of Uniform Multilayer Corrugated Shells

### 3.1. Process of the Explosion

**Figure 6:**
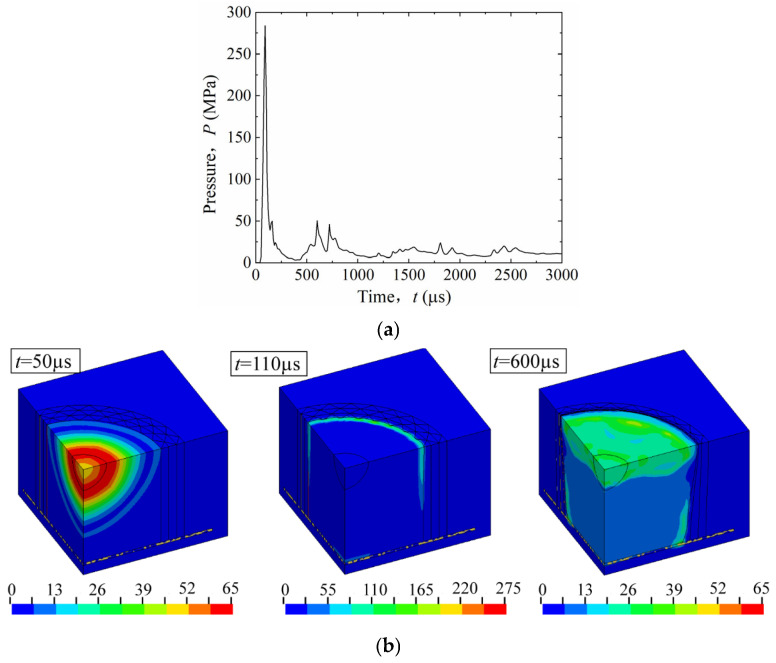
Process of explosion of 5 kg PE4 explosive within a closed uniform multilayer corrugated-core sandwich cylindrical shell: (**a**) pressure-time history; (**b**) pressure contour.

Based on the validated finite element model, this section investigates the response of the uniform multilayer corrugated-core sandwich cylindrical shell under internal blast loading. As shown in Figure 6, the blast load process is described through the pressure–time history and air-domain contours. Figure 6a shows the pressure–time history at an air element located on the Z-symmetry plane of the inner wall, while Figure 6b shows the corresponding pressure contours of the air domain. After detonation, the pressure wave initially propagated spherically into the surrounding space. At approximately 110 μs, the wave reached the inner wall along the radial direction, producing reflection and superposition, as shown in Figure 6b. This coincides with the first pressure peak in Figure 6a. The wave then propagated axially toward the rigid end plate, reflected, and interacted with the radial reflection (t ≈ 600 μs), creating the second and third pressure peaks. The simulated peak pressure was about 275 MPa, close to 292 MPa predicted by the Mills empirical formula [8], indicating that the FE simulation accurately reproduces the blast load. The Mills empirical formula is shown as follows:

(1) $$P=\frac{1772}{Z^{3}}-\frac{114}{Z^{2}}+\frac{108}{Z}$$

where $Z\;=\;\mathrm{stand}-\mathrm{off}\;\mathrm{distance}/\sqrt[{3}]{\mathrm{TNT}\;\mathrm{equivalent}\;}$, *P* is in kPa, and the TNT equivalent factor for PE4 is 1.3.

### 3.2. Dynamic Response of the Cylindrical Shell

**Figure 7:**
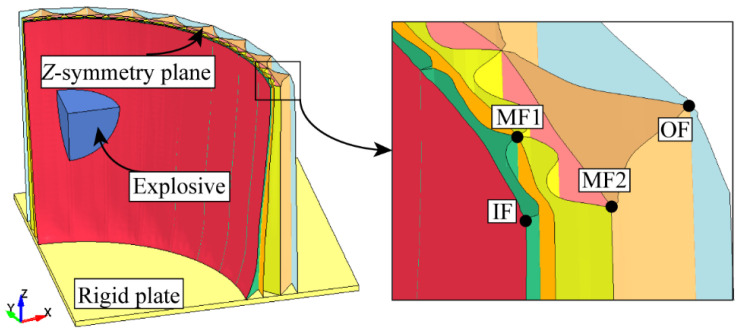
Configuration of the uniform multilayer corrugated-core sandwich cylindrical shells subjected to a 5 kg explosion.

Figure 7 shows the configuration of the uniform multilayer corrugated-core sandwich cylindrical shell (1/8 model) after the internal blast. The maximum compression of the corrugated core occurs at the Z-symmetry plane, which is closest to the explosive charge. From the Z-symmetry plane toward the rigid end plate, the compression gradually decreases due to the increasing stand-off distance and the plate constraint. The radial deformation of the facesheets also decreases from the innermost to the outermost layer (IF → OF) owing to core compression. At the Z-symmetry plane, four characteristic points were selected to evaluate the radial deformation of the inner facesheet (IF), intermediate facesheets (MF1, MF2), and outer facesheet (OF). Unless otherwise specified, the deformation data refer to these points. The radial deformation of the outer facesheet (OF) is used as the indicator for blast resistance performance: a smaller deformation implies stronger resistance.

**Figure 8:**
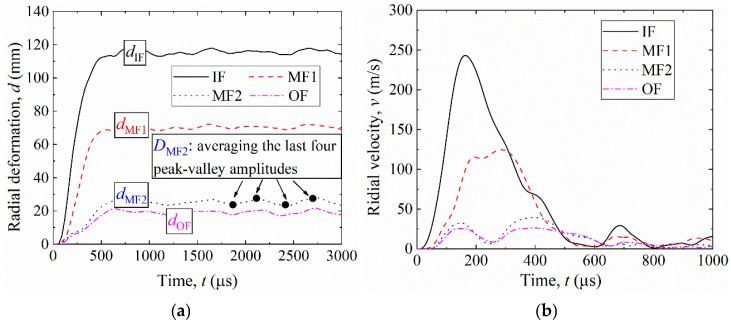
Dynamic response of the uniform multilayer corrugated-core sandwich cylindrical shell under a 5 kg explosion: (**a**) radial deformation curves; (**b**) radial-velocity curves.

Figure 8a shows the radial deformation–time histories, and Figure 8b shows the corresponding deformation-velocity histories for each facesheet. Following detonation, the inner facesheet (IF) deformed first, followed sequentially by MF1, MF2, and OF. The radial displacement history at the selected point on each facesheet is denoted as d(t). As seen in Figure 8a, the IF curve exhibits the largest displacement level (and peak), followed by MF1, MF2, and OF.

To quantitatively evaluate the dynamic response, two parameters were extracted from d(t): the final deformation *D* and the maximum deformation velocity $v_{max}$. The final deformation *D* was determined from the stabilized portion of the displacement–time curve as the average of the last four peak-to-valley amplitudes, representing the residual deformation after the transient oscillations had largely decayed. The maximum deformation velocity $v_{max}$ was defined as the maximum time-derivative of the displacement history during the loading process, i.e., $v_{max}=max(dd/dt)$.

Using these definitions, the final radial deformations were obtained as: *D*_IF_ = 116 mm, *D*_MF1_ = 71 mm, *D*_MF2_ = 26 mm, and *D*_OF_ = 20 mm. As shown in Figure 8b, the maximum deformation velocity reached 248 m/s for IF, 125 m/s for MF1, 34 m/s for MF2, and 25 m/s for OF. These results indicate that both the deformation amplitude and the deformation velocity decreased progressively from the inner to the outer facesheet, reflecting the sequential attenuation of blast loading through the multilayer shell structure.

**Figure 9:**
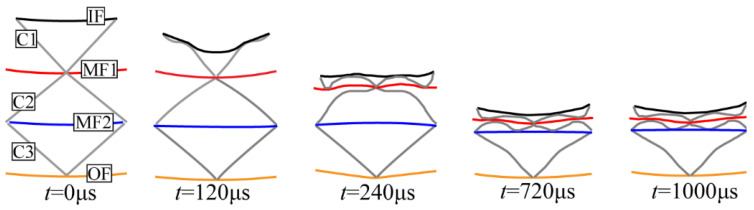
Deformation process of the uniform multilayer corrugated-core sandwich cylindrical shell under internal blast loading. Colors: IF (black), MF1 (red), MF2 (blue), OF (yellow), and corrugated core (gray).

To further illustrate the deformation mechanism, Figure 9 shows typical structural deformation profiles over time. A single unit cell was extracted for clarity, including the inner facesheet (IF), the three corrugated cores (C1–C3), and the intermediate and outer facesheets (MF1, MF2, OF). At *t* = 120 μs, IF dented inward and compressed C1. As C1 compacted, C2 and C3 began to deform (t = 240–720 μs), while MF1 and MF2 experienced in-plane stretching and bending. At *t* = 1000 μs, the deformation stabilized. Among all facesheets, IF deformed first and most severely, followed by MF1, then MF2 and OF, with smaller magnitudes. This sequence matches the data in Figure 8.

**Figure 10:**
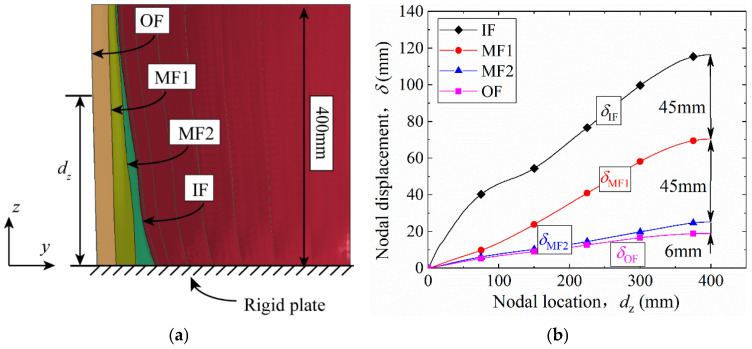
Deformation of each facesheet after stabilization: (**a**) generatrix nodal displacement; (**b**) variation of nodal displacement *δ* with nodal location *d*_z_ from the rigid end plate.

Figure 10 illustrates the generatrix deformation of each facesheet after stabilization (*t* = 3000 μs). Figure 10a shows the generatrix profiles, and Figure 10b shows the variation of nodal displacement *δ* with distance *d*_z_ from the rigid end plate. For all facesheets, *δ* increases with *d*_z_. At any given *d*_z_, *δ*_IF_ > *δ*_MF1_ > *δ*_MF2_ > *δ*_OF_. An inflection point at *d*_z_ = 80 mm on the IF curve results from the reflection and superposition of the pressure wave near the cylinder bottom, as discussed in Figure 6. As shown in Figure 10b, at *d*_z_ = 400 mm, the difference in nodal displacement (*δ*) between adjacent facesheets represents the maximum compression of the corrugated core layer between them. For example, the *δ* difference between the inner facesheet (IF) and the first middle facesheet (MF1) at *d*_z_ = 400 mm is 45 mm, indicating that the maximum compression of the first core (C1) is 45 mm. Similarly, the maximum compressions of the second (C2) and third (C3) corrugated cores are 45 mm and 6 mm, respectively. It can be observed that C1 and C2 undergo significantly greater compression than C3.

## 4. Blast Resistance of Graded Multilayer Structures with Varied Core Wall Thickness

In the uniform multilayer corrugated-core sandwich cylindrical shell, the third core layer (C3) experienced very limited compaction, while the outer facesheet (OF) underwent significant radial deformation. This non-uniform deformation indicates that the uniform configuration cannot effectively utilize the energy-absorbing capacity of its core layers under blast loading. To address this issue, a graded design was introduced by varying the wall thickness of the corrugated cores. Six graded configurations (Table 1) were analyzed under the same blast-loading conditions as the uniform shell in order to explore their blast-resistance performance.

### 4.1. Radial Deformation Behavior of Thickness-Graded Structures

**Figure 11:**
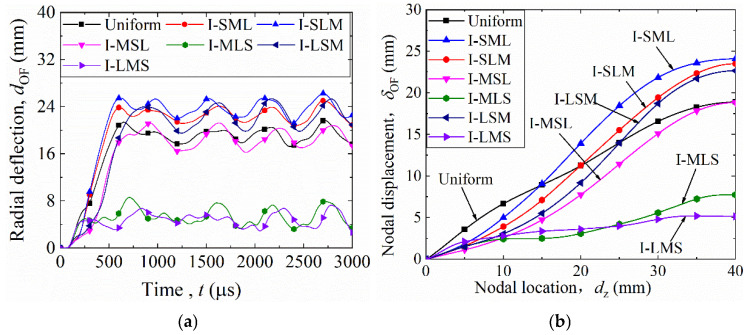
Radial deformation of the outer facesheet (OF) in uniform and thickness-graded multilayer sandwich cylindrical shells: (**a**) time-radial deformation curves; (**b**) nodal displacement (*δ*_OF_) along the generatrix.

Figure 11a shows the time-history curves of OF radial deformation (*t–d*_OF_) for the uniform and six graded configurations. After detonation, all curves rapidly reached their peaks and then oscillated. Configurations I-LMS and I-MLS exhibited the smallest deformation peaks, whereas the uniform shell and the other four graded models showed larger amplitudes. The average value of the last four oscillation peaks was taken as the final radial deformation (*D*_OF_), summarized in Table 8. The descending order of *D*_OF_ is: I-SLM > I-SML > I-LSM > uniform > I-MSL > I-MLS > I-LMS. Compared with the uniform shell (20 mm), the *D*_OF_ of I-LMS decreased to 5 mm (a 75% reduction). Therefore, the I-LMS configuration (with core wall thickness decreasing gradually from the inner to the outer layer, as shown in Table 1) exhibits the smallest radial deformation of the outer facesheet (OF) and the best blast resistance.

Figure 11b presents the OF nodal displacement (*δ*_OF_) along the generatrix (*d*_z_) after deformation stabilized. For *d*_z_ < 100 mm, the uniform shell had the largest *δ*_OF_, while I-MSL had the smallest. *δ*_OF_ increased with *d*_z_ for all configurations. Among them, I-MLS and I-LMS maintained the lowest *δ*_OF_ values and the flattest *δ*_OF_–*d*_z_ curves, indicating uniform and minimal deformation. Thus, the graded structure with a decreasing core wall thickness from inner to outer layers (I-LMS) exhibited the smallest overall deformation.

### 4.2. Mechanism of Improved Blast Resistance in Thickness-Graded Structures

**Figure 12:**
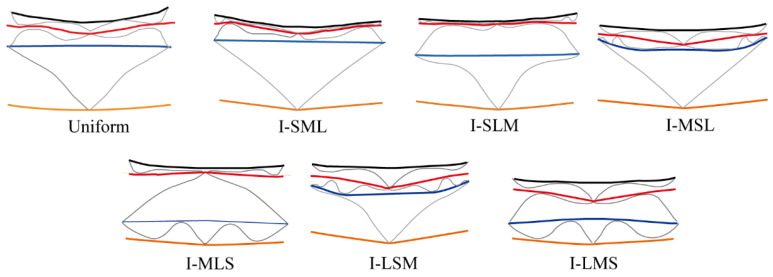
Collapse mode of thickness-graded sandwich cylindrical shells. Colors: IF (black), MF1 (red), MF2 (blue), OF (yellow), and corrugated core (gray).

To clarify the origin of the improved blast resistance, Figure 12 shows the final deformation profiles (*t* = 3000 μs) for the uniform and graded shells. Each structure contains IF, C1, MF1, C2, MF2, C3, and OF. Deformation of C3 was negligible in all configurations except I-MLS and I-LMS. In I-SML and I-MSL, C3 was almost undeformed. Conversely, C1 was fully compacted in most configurations except I-LSM and I-LMS. Among them, I-LMS showed the most uniform and complete compaction of the three cores.

These results are quantified in Table 8, where the core compaction ratio (*η*) is introduced to characterize the compressive deformation of each core layer under blast loading. For an individual core layer, the compaction ratio is defined as the reduction in layer height normalized by the initial layer height, i.e., *η* = (*h*_0_ − *h*)/*h*_0_, where *h*_0_ and *h* are the initial and deformed heights of the corresponding core layer, respectively. The overall compaction ratio is defined based on the total core height, i.e., *η*_overall_ = (*H*_0_ − *H*)/*H*_0_, where *H*_0_ and *H* are the initial and deformed total core heights. A larger η therefore indicates greater compression and densification of the corrugated core, whereas a smaller *η* indicates limited compression.

According to Table 8, I-LMS exhibits the highest overall core compaction, with *η*_overall_ = 0.67 and the largest local compaction in the C3 layer (*η* = 0.78). In contrast, I-SML and I-MSL show very limited compaction in the same layer, with *η* values of 0.06 and 0.05, respectively. Other configurations display non-uniform compaction, with some layers being nearly fully compacted (*η* > 0.8) while others remain only slightly compressed (*η* < 0.2). Overall, I-LMS demonstrates the greatest overall compaction and a comparatively more even compaction distribution among the three core layers.

Based on the analysis of the corrugated core compression process, the superior blast resistance of the I-LMS configuration can be explained as follows. In both the uniform and graded structures, the three corrugated core layers are compressed sequentially as the shock wave propagates outward and gradually attenuates. The core arrangement of I-LMS matches this attenuation process. The thickest-walled core is placed on the inner side, where the blast load is strongest, preventing full compaction. The thinnest-walled core is located on the outer side, where the transmitted load is weakest, allowing full compression. This design leads to the highest overall core layer compaction ratio and improved cushioning, thereby minimizing the radial deformation of the outer facesheet.

In contrast, the I-SML configuration places the thinnest-walled core nearest the explosion, causing immediate full compaction and poor cushioning. The thickest core, located outward, experiences too low a load to deform (C3 compaction ≈ 0.06). This imbalance limits energy absorption and increases OF deformation. Hence, I-SML demonstrates the poorest blast resistance among all configurations.

## 5. Blast Resistance of Graded Multilayer Structures with Varied Core Layer Height

This section studies graded designs obtained by changing the height of the corrugated core layers. The aim is to examine how core-height variation affects the blast resistance of multilayer corrugated sandwich cylindrical shells. Six height-graded configurations (Table 2) were analyzed under the same blast-loading conditions as in Section 4.

### 5.1. Radial Deformation Behavior of Height-Graded Structures

**Figure 13:**
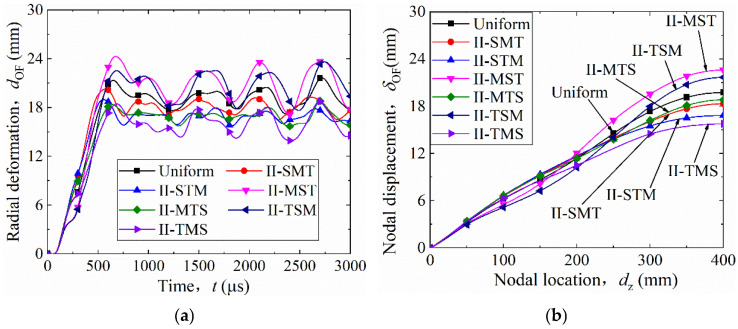
Radial deformation of the outer facesheet (OF) in uniform and height-graded multilayer sandwich cylindrical shells: (**a**) time-radial deformation curves; (**b**) nodal displacement (*δ*_OF_) along the generatrix.

Figure 13a shows the time-history curves of the outer facesheet (OF) radial deformation (*t*–*d*_OF_) for the uniform shell and six height-graded configurations. After detonation, all curves rise quickly to their peak values and then start to oscillate. The oscillation frequencies were similar in all cases. Larger *d*_OF_ values corresponded to larger oscillation amplitudes. Unlike the thickness-graded results, the *t*–*d*_OF_ curves of the height-graded structures were close to each other. No configuration showed a curve that was clearly lower than the others. Among them, structure II-TMS had the smallest *t*–*d*_OF_ curve, and structure II-MST had the largest.

The final radial deformation (*D*_OF_) was obtained by averaging the last four peaks of the *t*–*d*_OF_ curves. The results are listed in Table 9. The descending order of *D*_OF_ at the Z-symmetry plane is II-MST, II-TSM, Uniform, II-SMT, II-MTS, II-STM, and II-TMS. Compared with the uniform structure (20 mm), the *D*_OF_ of II-TMS was reduced to 15 mm (a 25% decrease). Therefore, the II-TMS, whose core layer height decreases gradually from the inner to the outer layer (Table 2), shows the smallest radial deformation of the outer facesheet and the best blast resistance. However, the effect is less pronounced than that of the thickness-graded model (I-LMS), which reduced *D*_OF_ by 75%. The benefit from height gradient is therefore less significant than from thickness grading.

Figure 13b shows the nodal displacement (*δ*_OF_) along the generatrix (*d*_z_) after deformation stabilized. For all configurations, *δ*_OF_ increased with *d*_z_. When *d*_z_ < 200 mm, the curves of different models were very close, showing similar deformation near the fixed end. When *d*_z_ > 200 mm, their differences became larger. Beyond *d*_z_ = 300 mm, the order of the curves no longer changed. From highest to lowest *δ*_OF_, the order was II-MST, II-TSM, Uniform, II-SMT, II-MTS, II-STM, and II-TMS. This shows that near the symmetry plane (*d*_z_ < 200 mm), all models behaved similarly, but farther away (*d*_z_ > 300 mm), structure II-TMS had the smallest outer-facesheet deformation.

### 5.2. Mechanism of Improved Blast Resistance in Height-Graded Structures

**Figure 14:**
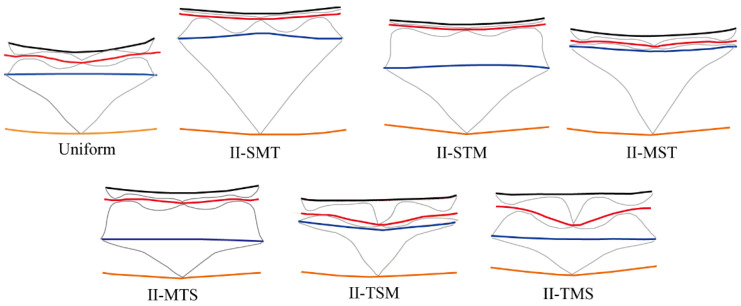
Collapse mode of height-graded multilayer sandwich cylinders. Colors: IF (black), MF1 (red), MF2 (blue), OF (yellow), and corrugated core (gray).

Among the uniform and graded models, II-TMS showed the smallest final radial deformation and the least overall deformation of the outer facesheet. To explain this result, Figure 14 shows the final deformation profiles (*t* = 3000 µs) of all configurations. Each model includes the inner facesheet (IF), three corrugated cores (C1–C3), two middle facesheets (MF1 and MF2), and the outer facesheet (OF). Except for II-TSM and II-TMS, the first core (C1) was fully compacted in all models. In II-MST and II-TSM, the second core (C2) was compacted the most. The third core (C3) was highly compacted in II-MTS, II-TSM, and II-TMS, but only slightly compressed in other models. In II-SMT, C3 was almost undeformed.

The quantitative results are given in Table 9. Structure II-TMS shows the highest C3 compaction ratio (*η* = 0.27) and the highest overall compaction ratio (*η*_overall_ = 0.60) among the height-graded configurations. Other configurations exhibit non-uniform compaction, where some layers are highly compacted while others remain only slightly compressed. Therefore, II-TMS achieves a comparatively more balanced compaction distribution across the three core layers, which contributes to its improved blast resistance within the height-graded group. However, the C3 compaction in II-TMS (*η* = 0.27) is still much lower than that of the thickness-graded I-LMS configuration (*η* = 0.78), which explains why the blast resistance of II-TMS remains inferior to that of I-LMS.

## 6. Parametric Analysis and Optimization

In the previous sections, the explosive mass and the differences in wall thickness or height among the core layers were predetermined. This section performs a parametric analysis to examine how variations in explosive mass and gradient magnitude affect the radial deformation of the structure. Subsequently, a surrogate-based optimization method is used to identify the optimal core wall thickness configuration within specified limits.

### 6.1. Parametric Analysis

In this section, thickness- and height-gradient structures follow the same basic pattern: the inner layer is the thickest (or tallest), and the outer layer is the thinnest (or shortest).

#### 6.1.1. Core-Thickness-Graded Structures

For the thickness-graded model, the wall thickness difference between adjacent corrugated cores is denoted as *t*_d_. This difference remains constant across both layer interfaces, such that *t*_d_ = *t*_c1_ − *t*_c2_ = *t*_c2_ − *t*_c3_. The middle-core thickness was fixed at *t*_c2_ = 2 mm, and three gradient cases were created with *t*_d_ = 0.5 mm, 1.0 mm, and 1.5 mm. A larger *t*_d_ represents a steeper gradient. The corresponding structures are named I-LMS-0.5, I-LMS-1.0, and I-LMS-1.5, respectively. The response of each structure was studied under PE4 explosive masses ranging from 1 kg to 7 kg in 1 kg increments.

**Figure 15:**
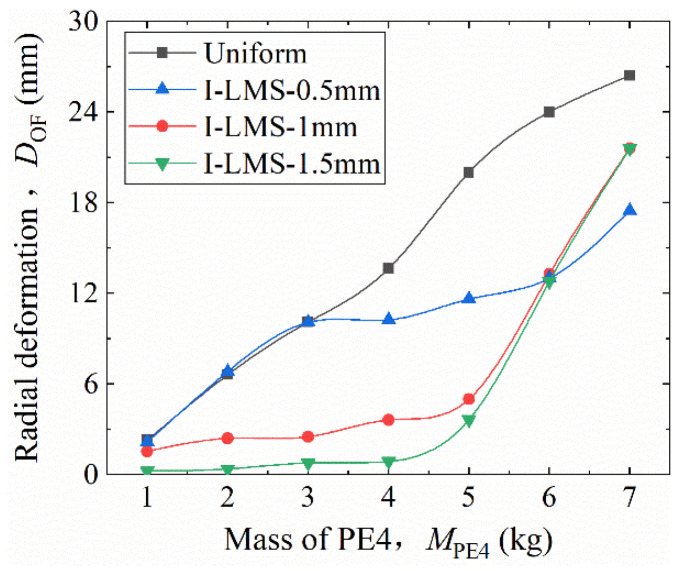
Parametric analysis of radial deformation in thickness-graded configurations under blast loading.

The variation of the final radial deformation (*D*_OF_) with explosive mass (*M*_PE4_) is shown in Figure 15. The following observations can be made: (i) As *M*_PE4_ increases, the *D*_OF_ of all configurations grows; (ii) when *M*_PE4_ ≤ 3 kg, I-LMS-1.5 exhibits the smallest *D*_OF_, while I-LMS-0.5 shows no advantage over the uniform structure. Under these conditions, structures with steeper gradients achieve better blast resistance. (iii) When 3 kg ≤ *M*_PE4_ ≤ 5 kg, I-LMS-0.5 begins to show an advantage, and the order of *D*_OF_ (from largest to smallest) becomes: uniform > I-LMS-0.5 > I-LMS-1.0 > I-LMS-1.5. A steeper gradient still provides better resistance. (iv) When *M*_PE4_ *≥* 5 kg, *D*_OF_ continues to increase for all configurations, but I-LMS-0.5 and I-LMS-1.0 grow more slowly than I-LMS-1.5. The advantage of steep gradients decreases, and a milder gradient then yields better resistance.

Thus, core-thickness-graded structures consistently outperform the uniform one, but their advantage decreases at higher blast loading. Under weaker explosions, a steeper gradient is more effective; under stronger blasts, a milder gradient performs better.

#### 6.1.2. Core-Height-Graded Structures

For the height-graded model, the height difference between adjacent corrugated cores is denoted as *h*_d_. This difference also remains constant, satisfying *h*_d_ = *h*_c1_ − *h*_c2_ = *h*_c2_ − *h*_c3_. The height of the middle core was fixed at *h*_c2_ = 30 mm, and three gradient cases were constructed with *h*_d_ = 5 mm, 10 mm, and 15 mm. Again, a larger *h*_d_ value indicates a steeper gradient. The corresponding structures are named II-TMS-5, II-TMS-10, and II-TMS-15. As with the thickness-graded models, each configuration was subjected to PE4 explosive masses from 1 kg to 7 kg.

**Figure 16:**
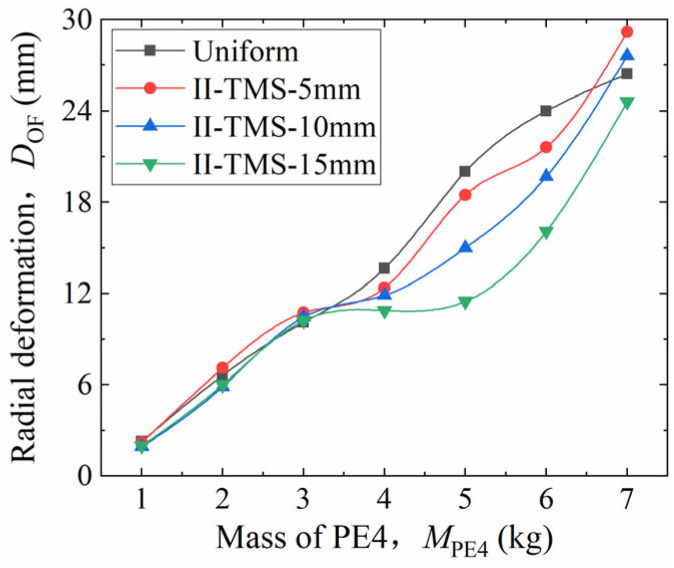
Parametric analysis of radial deformation in height-graded configurations.

The resulting radial deformation (*D*_OF_) values are presented in Figure 16. It was shown that: (i) when *M*_PE4_ ≤ 3 kg, *D*_OF_ values of the uniform and graded shells are nearly equal, showing no significant advantage from the height gradient; (ii) when 3 kg ≤ *M*_PE4_ ≤ 7 kg, the graded shells outperform the uniform shell. The order of decreasing *D*_OF_ is: uniform > II-TMS-5 > II-TMS-10 > II-TMS-15. A steeper height gradient (larger *h*_d_) results in smaller deformation and improved blast resistance.

In summary, for weaker blasts, height grading has little effect. As the blast load increases, steeper height gradients improve the structural performance. Overall, the effect of height grading is weaker than that of thickness grading, but it still enhances the blast-resistance property when properly matched to the blast intensity.

### 6.2. Optimization

The preceding analysis shows that adopting a core thickness gradient that gradually decreases from the inner to the outer layer can effectively reduce the radial deformation of cylindrical shells under internal blast loading and significantly enhance their blast resistance. However, due to computational limitations, the previous study examined only a few discrete gradient cases and assumed identical thickness differences between adjacent core layers. This simplified setting may overlook the truly optimal gradient configuration.

**Figure 17:**
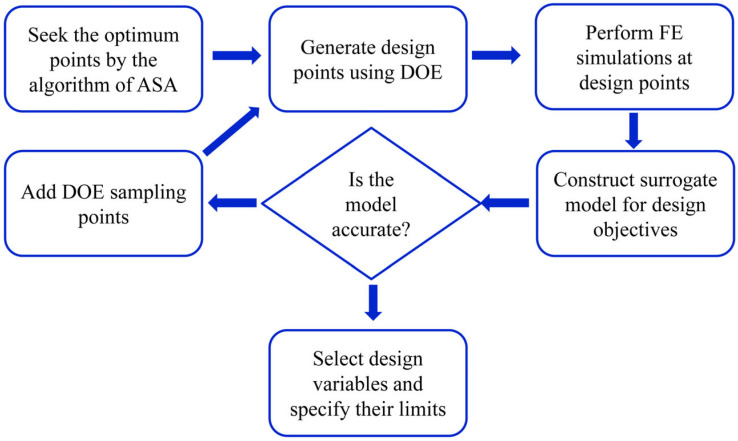
Flow chart of the surrogate-based optimization process.

To identify the most efficient design, a surrogate-based optimization method is employed in this section. The optimization determines the best combination of core wall thicknesses within the specified range under a 5 kg blast load. The main steps of the optimization procedure are illustrated in Figure 17, and the corresponding methods and results are presented in the following subsections.

#### 6.2.1. Definition of Optimization Problem

Two independent optimization problems were proposed, as summarized in Table 10. The first optimization problem (Opt. 1) focuses on lightweight structural design. It aims to determine the minimum mass configuration under a specified constraint on the radial deformation (*D*_OF_). The design variables are the wall thicknesses of the three corrugated cores. The upper limit of *D*_OF_ is set to 5 mm, according to the result obtained from the I-LMS-1.0 configuration. The thicknesses of the three cores are allowed to vary between 1 mm and 3 mm. The second optimization problem (Opt. 2) focuses on enhancing blast resistance. It seeks the configuration with the smallest radial deformation under a prescribed structural mass (*M*_s_). The design variables are again the wall thicknesses of the three corrugated cores, which are allowed to vary between 1 mm and 3 mm. The upper limit for the total structural mass (*M*_s_) was set to 30.55 kg, corresponding to the mass of the I-LMS-1.0 configuration.

#### 6.2.2. Surrogate Model

A total of 89 design points were generated using the Optimal Latin Hypercube (OLH) sampling method, as listed in Appendix A. Based on the finite element simulation results for these points, a Radial Basis Function (RBF) model was constructed to fit the relationship between the design variables and the response quantities. Cross-validation analysis was employed to evaluate model accuracy. The coefficient of determination (*R*^2^), the Root Mean Square Error (RMSE), and the Maximum Absolute Percentage Error (MAPE) were calculated, as given by Equations (2)–(4):

(2) $$R^{2}=1-\frac{\sum_{i=1}^{N}{\left(y_{i}-{\hat{y}}_{i}\right)}^{2}}{\sum_{i=1}^{N}{\left(y_{i}-{\bar{y}}_{i}\right)}^{2}}$$

(3) $$\mathrm{RSME}=\sqrt{\frac{1}{N}\sum_{i=1}^{N}\left({\hat{y}}_{i}-y_{i}\right)}$$

(4) $$\mathrm{MAPE}=\max\left(\frac{\left|{\hat{y}}_{i}-y_{i}\right|}{y_{i}}\right)$$

where $y_{i}$, ${\bar{y}}_{i}$, ${\hat{y}}_{i}$ and *N* represent the actual finite element results, the RBF-predicted values at the validation points, and the total number of validation samples, respectively.

**Figure 18:**
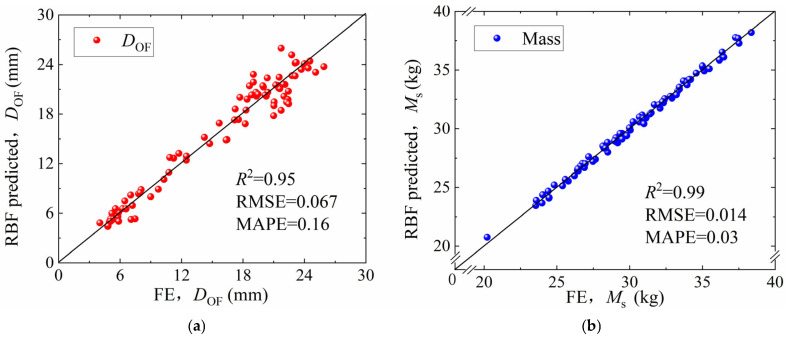
Cross-validation results of the RBF model: (**a**) final radial deformation; (**b**) structural mass.

Figure 18 compares the RBF model predictions and the finite element calculation results, along with their corresponding values of *R*^2^, RMSE, and MAPE. The relative errors of the surrogate model prediction for both the final radial deformation (*D*_OF_) and the structural mass (*M*_s_) are within acceptable ranges. In all cases, the coefficient of determination R^2^ exceeds 0.95, indicating that the RBF surrogate model provides sufficient prediction accuracy.

#### 6.2.3. Optimization Results

Both optimization problems were solved using the Adaptive Simulated Annealing (ASA) algorithm. This method is suitable for highly nonlinear engineering problems because it can effectively locate global optimal solutions with relatively low computational cost [41].

The optimization results are summarized in Table 11. The table lists the optimized wall thicknesses (*t*_c1_–*t*_c3_), the final radial deformation (*D*_OF_), and the structural mass (*M*_s_). Corresponding finite element results are also provided for verification. The differences between the finite element and optimization results are small, confirming the reliability of the surrogate model and the optimization process. For comparison, the table also includes the finite element results of the uniform multilayer corrugated shell, the I-LMS-1.0 configuration, and a single-wall cylindrical shell of equivalent mass.

The first optimization problem (Opt. 1) aims to minimize the structural mass *M*_s_ while keeping the radial deformation *D*_OF_ not greater than 5 mm. The optimized result suggests a core thickness distribution that gradually decreases from inner to outer layers, in agreement with the I-LMS-1.0 configuration. However, the thickness differences between adjacent layers are not constant: *t*_c1_ − *t*_c2_ = 0.9 mm and *t*_c2_ − *t*_c3_ = 0.3 mm. This indicates a larger thickness difference between C1 and C2 and a smaller difference between C2 and C3. In terms of performance, the optimized structure achieves an 18% reduction in *M*_s_ while maintaining the same *D*_OF_ as the I-LMS-1.0 configuration. Compared with the uniform multilayer shell, the optimized structure reduces *M*_s_ by 19% and *D*_OF_ by 75%. Relative to the single-wall shell of equivalent mass, *M*_s_ decreases by 19% and *D*_OF_ decreases by 77%.

The second optimization problem (Opt. 2) focuses on maximizing blast resistance by minimizing *D*_OF_ under the constraint of equal total mass (*M*_s_ ≤ 30.55 kg). The optimized result again follows the pattern of decreasing thickness from inner to outer layers, similar to the I-LMS-1.0 and Opt. 1 configurations, but with non-uniform interlayer differences: *t*_c1_ − *t*_c2_ = 0.1 mm, *t*_c2_ − *t*_c3_ = 1.0 mm. This indicates a small gradient between C1 and C2 and a larger gradient between C2 and C3. The optimized structure reduces *D*_OF_ by 20% while maintaining nearly the same *M*_s_ as I-LMS-1.0. Compared with the uniform multilayer shell under equal mass, it reduces *D*_OF_ by 80%, and compared with the single-wall shell of equivalent mass, by 81%.

In summary, both optimization methods markedly improve the structural efficiency and blast resistance performance. The optimal configurations all feature gradually decreasing wall thicknesses from *t*_c1_ to *t*_c3_, consistent with the inner-to-outer reduction pattern identified earlier. However, unlike the previous parametric designs, the optimal structures exhibit non-constant thickness differences between adjacent core layers. The two optimization schemes lead to different gradient patterns, depending on whether the objective is lightweight design (Opt. 1) or blast-resistance enhancement (Opt. 2).

## 7. Conclusions

This study proposes a graded multilayer corrugated sandwich cylindrical shell to improve resistance against internal blast loading. Finite element analyses were conducted on uniform, core-thickness-graded, and core-height-graded structures, followed by a surrogate-based optimization study. The main findings are summarized as follows:

(1) For the uniform multilayer shell, the core layers collapse sequentially from the inner to the outer side. The inner cores (C1 and C2) are highly compacted, whereas the outer core (C3) shows limited compaction, indicating that the outer core is not fully utilized.
(2) Gradient designs significantly enhance blast resistance within the investigated range (PE4: 1–7 kg). The core-thickness-graded configuration provides larger reductions in the outer-facesheet deformation than both the uniform and the core-height-graded designs.
(3) The improved performance is linked to the outward attenuation of blast pressure: by assigning higher core strength to the inner side and lower strength to the outer side, the three core layers compact more evenly and more completely, which reduces the final radial deformation of the outer facesheet.
(4) For the thickness-graded designs, the outer-facesheet final radial deformation (*D*_OF_) increases with the PE4 mass (*M*_PE4_), but the best gradient magnitude changes with charge level: I-LMS-1.5 performs best up to 3 kg; from 3 to 5 kg, *D*_OF_ decreases from the uniform design to I-LMS-0.5 and then to I-LMS-1.0, with I-LMS-1.5 remaining the smallest; above 5 kg, I-LMS-0.5 and I-LMS-1.0 show a slower increase in *D*_OF_ than I-LMS-1.5.
(5) Surrogate-based optimization yields both lightweight and blast-resistant configurations. The optimal designs feature an inner-to-outer decrease in wall thickness with non-uniform layer differences. From a practical design viewpoint, key factors to focus on include the expected loading intensity, the overall gradient of the core wall thickness, the thickness ratio between layers, and the balance between weight and allowable deformation.

These findings provide practical guidance for designing graded corrugated-core sandwich cylindrical shells under internal blast loading.

## Figures and Tables

**Table 1:** Configurations of Type I graded sandwich cylindrical shells with varying core wall thickness.

Structure	Thickness			Mass/kg
	*t*_c1_/mm	*t*_c2_/mm	*t*_c3_/mm	
Uniform	2	2	2	30.85
I-SML	1	2	3	31.15
I-SLM	1	3	2	31.85
I-MSL	2	1	3	30.14
I-MLS	2	3	1	31.55
I-LSM	3	1	2	29.85
I-LMS	3	2	1	30.55

**Table 2:** Configurations of Type II graded sandwich cylindrical shells with varying core height.

Structure	Height			Mass/kg
	*h*_c1_/mm	*h*_c2_/mm	*h*_c3_/mm	
Uniform	30	30	30	30.85
II-SMT	20	30	40	30.56
II-STM	20	40	30	30.77
II-MST	30	20	40	30.77
II-MTS	30	40	20	31.18
II-TSM	40	20	30	31.18
II-TMS	40	30	20	31.39

**Table 3:** Material properties of 304 stainless steel. Reprinted with permission from Ref. [8]. Copyright 2014 Elsevier.

*ρ* (kg/m^3^)	*G* (GPa)	*E* (GPa)	*A* (MPa)	*B* (MPa)	*n*	*D* (s^−1^)	*q*	*T*_melt_ (K)	*T*_room_ (K)
7900	81.8	200	310	1015	0.59	100	10	1673	298

**Table 4:** Parameters for the EOS_GRUNEISEN of 304 stainless steel. Reprinted with permission from Ref. [8]. Copyright 2014 Elsevier.

*C*_o_ (m/s)	*S*_1_	*S*_2_	*S*_3_	γ_o_
4578	1.49	0	0	1.93

**Table 5:** Material and equation of state parameters for the PE4 explosive. Reprinted with permission from Ref. [8]. Copyright 2014 Elsevier.

*ρ* (kg/m^3^)	*D* (m/s)	PCJ (GPa)	*A*	*B*	*R*_1_	*R*_2_	*ω*	*e*_o_ (MJ/m^3^)
1601	8193	28	609.77	12.95	4.5	1.4	0.25	9000

**Table 6:** Material and equation of state parameters for air. Reprinted with permission from Ref. [8]. Copyright 2014 Elsevier.

*ρ* (kg/m^3^)	*e*_o_ (kJ/kg)	*C*_1_	*C*_2_	*C*_3_	*C*_4_	*C*_5_	*C*_6_
1.184	253.3	0	0	0	0.4	0.4	0

**Table 7:** Comparison of experimental and numerical maximum radial deformations for PE4 explosive charges (25–55 g) [8].

PE4 Charge (g)	Experimental Results [8] (mm)	3D FE Results (mm)	2D FE Results [8] (mm)	Experiment vs. 3D FE
25	3.2	3.4	3.0	6.2%
30	3.2	4.76	4.3	48.8%
35	6.6	6.2	5.6	−6.1%
40	7.1	7.23	7.0	1.8%
45	8.5	9.05	8.8	6.5%
50	11.5	10.89	11.0	−5.3%
55	14.2	12.68	12.4	−10.7%

**Table 8:** Compaction ratio, deformation and mass of thickness-graded cylindrical shells.

Structure	Compaction Ratio, *η*				Radial Deflection (mm)	Mass (kg)
	C1	C2	C3	Overall		
Uniform	0.76	0.75	0.11	0.54	20	30.85
I-SML	0.85	0.85	0.06	0.59	24	31.15
I-SLM	0.95	0.45	0.15	0.52	23	31.85
I-MSL	0.73	0.97	0.05	0.62	19	30.14
I-MLS	0.95	0.17	0.76	0.62	8	31.55
I-LSM	0.59	0.96	0.09	0.56	23	29.85
I-LMS	0.65	0.59	0.78	0.67	5	30.55

**Table 9:** Compaction ratio, displacement and mass of height-graded cylindrical shells.

Structure	Compaction Ratio, *η*				Radial Deflection (mm)	Mass (kg)
	C1	C2	C3	Overall		
Uniform	0.76	0.75	0.11	0.54	20	30.85
II-SMT	0.91	0.60	0.03	0.42	18	30.56
II-STM	0.91	0.45	0.06	0.42	17	30.77
II-MST	0.76	0.95	0.11	0.53	23	30.77
II-MTS	0.80	0.58	0.19	0.57	19	31.18
II-TSM	0.66	0.97	0.09	0.57	22	31.18
II-TMS	0.65	0.74	0.27	0.60	15	31.39

**Table 10:** Definition of the objective optimization problems.

Case	Objective	Constraint	Variable
1	Minimize *M*_s_	*D*_OF_ ≤ 5 mm 1 mm≤ *t*_c1_ ≤3 mm 1 mm≤ *t*_c2_ ≤3 mm 1 mm≤ *t*_c3_ ≤3 mm	*t*_c1_ *t*_c2_ *t*_c3_
2	Minimize *D*_OF_	*M*_s_ ≤ 30.55 kg 1 mm≤ *t*_c1_ ≤3 mm 1 mm≤ *t*_c2_ ≤3 mm 1 mm≤ *t*_c3_ ≤3 mm	*t*_c1_ *t*_c2_ *t*_c3_

**Table 11:** Comparison between FE simulation results and optimization solutions.

Specimen	*t*_c1_	*t*_c2_	*t*_c3_	*M* (kg)		*D* (mm)	
				Opt	FE	Opt	FE
Opt case1	2.2	1.3	1	24.30	24.91	5	5
Opt case2	2.3	2.2	1	29.09	28.98	4	4
I-LMS	3	2	1	30.55		5	
Uniform	2	2	2	30.85		20	

## Data Availability

The original contributions presented in this study are included in the article. Further inquiries can be directed to the corresponding author.

## Appendix A

**Table A1:** Sampling points and numerical results at these points.

Number	*t_c_*_1_ (mm)	*t_c_*_2_ (mm)	*t_c_*_3_ (mm)	*D*_OF_ (mm)	*M* (kg)
1	2.56	2.80	1.34	7.21	33.43
2	1.37	1.44	2.86	18.41	29.49
3	2.97	2.12	2.63	18.85	36.17
4	2.32	2.19	1.54	9.74	30.95
5	1.10	2.63	2.56	23.10	32.40
6	1.71	1.31	1.95	21.00	26.95
7	1.48	2.42	2.19	24.00	31.49
8	2.73	2.05	2.05	19.39	33.30
9	1.17	1.98	2.73	21.20	30.98
10	1.03	2.70	1.75	17.71	29.80
11	1.31	1.92	1.10	5.55	25.39
12	2.86	1.51	2.22	19.02	32.09
13	2.19	1.71	1.24	5.80	27.66
14	1.54	1.88	2.02	20.38	28.97
15	2.25	1.27	2.15	18.64	29.11
16	2.53	2.90	2.49	24.53	37.51
17	1.75	1.41	1.03	5.21	24.46
18	2.80	1.61	2.80	17.25	34.19
19	2.12	2.66	2.83	23.10	36.47
20	2.46	2.32	2.53	21.53	35.13
21	1.81	1.58	2.46	20.16	30.01
22	1.85	2.46	1.78	18.23	31.40
23	2.83	2.86	1.85	17.18	36.18
24	1.68	1.68	1.51	10.85	26.89
25	2.70	2.59	2.97	21.60	38.36
26	2.59	1.64	1.68	11.24	30.05
27	2.29	2.29	1.00	4.03	29.48
28	1.44	2.97	2.09	22.77	33.21
29	2.09	1.81	1.92	18.30	29.99
30	1.14	1.17	1.88	19.02	24.47
31	1.07	1.75	1.64	17.56	25.79
32	1.00	2.09	2.12	23.20	28.50
33	1.92	1.07	2.70	20.00	29.07
34	2.05	3.00	1.71	14.23	33.95
35	1.41	1.03	2.39	21.03	26.41
36	1.98	2.76	2.25	24.37	34.58
37	2.39	2.53	1.98	22.40	33.95
38	1.27	2.49	1.07	6.46	27.48
39	2.02	2.73	1.20	6.25	31.09
40	1.61	2.93	2.66	21.73	35.47
41	2.49	1.20	1.14	5.87	26.21
42	2.63	1.14	2.59	19.32	31.13
43	2.15	1.24	1.58	11.74	26.78
44	1.24	1.34	1.31	10.77	23.56
45	2.36	1.78	2.42	19.27	32.33
46	1.64	1.00	1.48	15.68	23.98
47	2.90	2.36	1.41	7.04	32.91
48	1.20	1.54	2.32	19.47	27.60
49	1.51	2.83	1.44	10.29	30.76
50	1.95	2.15	2.36	21.93	32.38
51	3.00	1.37	1.37	8.06	29.18
52	2.76	1.85	1.17	5.34	29.34
53	2.22	1.48	2.93	21.50	32.02
54	1.34	2.25	1.61	14.75	28.16
55	1.88	1.95	2.90	21.40	32.79
56	2.93	2.56	2.29	22.80	36.36
57	1.58	2.39	2.76	25.20	33.19
58	2.42	2.02	3.00	20.42	35.02
59	1.78	2.22	1.27	6.58	28.23
60	2.66	1.10	1.81	16.44	28.18
61	1.89	1.22	1.22	7.97	24.40
62	1.67	1.89	3.00	19.95	32.26
63	1.00	1.44	1.89	22.47	24.82
64	3.00	1.67	1.44	8.99	30.23
65	1.44	2.33	1.00	7.07	26.76
66	1.22	2.56	2.11	25.90	30.66
67	2.78	2.11	2.56	21.05	35.01
68	2.56	2.78	1.67	12.48	34.10
69	2.33	1.00	2.33	22.27	28.48
70	2.11	3.00	2.78	25.10	37.28
71	1.22	3.00	1.89	22.00	31.70
72	1.67	1.00	1.44	16.36	23.59
73	2.11	1.22	3.00	22.43	30.90
74	1.44	2.11	1.22	6.58	26.61
75	2.33	2.78	1.67	12.49	33.41
76	1.00	1.67	2.33	20.27	27.18
77	3.00	2.56	2.56	22.10	37.46
78	1.89	2.33	2.78	23.70	33.95
79	2.78	1.44	2.11	21.73	30.86
80	2.56	1.89	1.00	4.83	28.35
81	2.81	3.00	1.05	7.48	33.70
82	2.36	1.50	1.07	5.09	26.44
83	1.00	1.00	1.00	7.80	20.21
84	2.47	1.46	1.01	4.94	26.47
85	2.79	2.22	1.07	5.09	30.67
86	2.29	2.27	1.00	4.81	29.06
87	1.65	1.49	1.00	5.87	24.03
88	2.29	2.27	1.00	4.80	29.06
89	1.90	1.65	1.04	5.14	25.58
